# Relative contributions of statin intensity, achieved low-density lipoprotein cholesterol level, and statin therapy duration to cardiovascular risk reduction in patients with type 2 diabetes: population based cohort study

**DOI:** 10.1186/s12933-022-01466-z

**Published:** 2022-02-22

**Authors:** Ji Yoon Kim, Jimi Choi, Sin Gon Kim, Nam Hoon Kim

**Affiliations:** Division of Endocrinology and Metabolism, Department of Internal Medicine, Korea University College of Medicine, 73, Goryeodae-ro, Seongbuk-gu, Seoul, 02841 Republic of Korea

**Keywords:** Statin duration, Statin intensity, Low-density lipoprotein cholesterol, Cardiovascular risk, Type 2 diabetes mellitus

## Abstract

**Background:**

Current guidelines recommend life-long use of statin for patients with type 2 diabetes (T2D), however, a number of patients discontinue statin therapy in clinical practice. We aimed to estimate the optimal statin therapy including statin therapy duration, statin intensity, and low-density lipoprotein cholesterol (LDL-C) level among patients with T2D in a real-world setting.

**Methods:**

From Korean National Health Insurance Service Cohort (2007–2015), 8937 patients with T2D (≥ 40 years of age) who received statin therapy for at least 90 days were included. Risk of major adverse cardiovascular event (MACE) including ischemic heart disease, ischemic stroke, and cardiovascular death was estimated according to statin intensity, achieved serum LDL-C level, and statin therapy duration, respectively. The relative contributions of these factors to MACE risk were quantified by calculating the proportion of log-likelihood explained by each factor.

**Results:**

The hazard ratio (HR) of MACE was lower in patients receiving moderate- or high-intensity statins than in those receiving low-intensity statins (HR, 0.72; *p* = 0.027). Among patients who received moderate- or high-intensity statins, lower achieved LDL-C level was associated with lower cardiovascular risk. Notably, the longer the patients received statins, the lower was the risk of MACE; the HR of MACE was significantly reduced after at least 18 months (adjusted HR, 0.70; *p* = 0.009) as a reference to 3–6 months of therapy. The proportion of explainable log-likelihood for MACE was greatest for statin duration (2.55), followed by achieved LDL-C level (2.18), and statin intensity (0.95).

**Conclusions:**

Statin therapy duration is as important as or more crucial than statin intensity or achieved LDL-C level for the reduction of cardiovascular risk in T2D patients. The concept of “longer is better” regarding statin therapy should be considered in clinical practice.

**Supplementary Information:**

The online version contains supplementary material available at 10.1186/s12933-022-01466-z.

## Background

Type 2 diabetes (T2D) is frequently accompanied by dyslipidemia, which is characterized by increased triglyceride (TG), decreased high-density lipoprotein cholesterol (HDL-C), and increased small dense low-density lipoprotein cholesterol (LDL-C) particles [1, 2]. Previous studies reported that LDL-C lowering with statin therapy substantially reduces the risk of atherosclerotic cardiovascular events in patients with T2D [3–5], accordingly, current guidelines for dyslipidemia management generally recommend life-long use of moderate- or high-intensity statins in adult patients with T2D [6, 7].

However, debates persist regarding whether the use of statins is crucial or whether achieved LDL-C concentration determines the risk reduction of atherosclerotic cardiovascular disease. The so-called statin and LDL-C hypotheses have been supported by evidence provided by randomized controlled trials (RCT). However, these hypotheses are often not reproducible in population-based studies [8], probably due to biases that are difficult to control. One of the major biases is non-adherence to statin therapy in clinical practice. In a meta-analysis, half of the patients who newly received statins discontinued therapy within a year [9]. Therefore, adherence to statin therapy may be critical for explaining the cardiovascular benefits of statins in clinical practice. We noted that only a few studies regarding statin therapy duration have been conducted [10, 11]. In addition, the duration of follow-up in many previous RCT on statins was often not long enough to observe the life-long effects of statin therapy [12–14]. Moreover, despite the numerous trials of statins, there are only few studies targeted patients with T2D [4, 15].

This study aimed to estimate the optimal statin therapy, especially the duration of statin therapy, among patients with type 2 diabetes in a real-world setting. We also aimed to compare the relative importance of the components of statin therapy, including statin intensity, achieved LDL-C level, and statin therapy duration, for cardiovascular risk reduction.

## Methods

### Data source and patient selection

We used the Korean National Health Insurance Service-Health Screening Cohort (NHIS-HEALS). This database contains longitudinal (2002–2015) information of 514,866 Koreans, including individuals’ demographic details, disease diagnoses according to the International Classification of Disease, Tenth Revision (ICD-10), prescription records, medical procedures, hospitalizations, death records, and health examination data, including laboratory data, anthropometric measures, and questionnaires for medical conditions. This cohort represented 10% of all health screening participants aged 40–79 years randomly selected in South Korea. The protocols have been described previously [16].

A flow diagram of the study subject selection process is shown in Additional file 1: Fig. S1. From the original cohort, we first selected adults (> 40 years) diagnosed with T2D who had received statins for at least 90 days between January 1, 2007 and December 31, 2013 since serum lipid profiles were available from 2007. Subjects without documented lipid profiles before statin therapy initiation were excluded. T2D was defined based on ICD-10 codes of T2D (E11-14), with the use of any glucose-lowering agent, including insulin. Subjects who had changed statin intensity during follow-up (n = 425) and those who were not covered by regular medical insurance (n = 113) were excluded. Finally, 11,219 patients were included in the study. Among them, 383 received low-intensity statins and 10,836 received moderate- or high-intensity statins. Statin intensity was classified according to the 2013 American Heart Association/American College of Cardiology guidelines on the management of blood cholesterol [17]. The following are classified as low-intensity statins: Simvastatin 10 mg, Pravastatin 10–20 mg, Lovastatin 20 mg, Fluvastatin 20–40 mg, and Pitavastatin 1 mg. These other statins have been classified as moderate- or high-intensity statins: Atorvastatin 10–80 mg, Rosuvastatin 5–40 mg, Simvastatin 20–40 mg, Pravastatin 40–80 mg, Lovastatin 40 mg, Fluvastatin XL 80 mg or 40 mg twice daily, and Pitavastatin 2–4 mg.

To assess the effects of statin intensity on cardiovascular risk reduction, propensity score matching (maximum 1:27) was performed for those who received low-intensity statins and those who received moderate- or high-intensity statins to balance covariates between groups. The propensity score model was derived from a multiple logistic regression that included age, sex, pre-existing cardiovascular disease (CVD), smoking status (never, former, or current), alcohol consumption (never, ≤ 2 times/week, or ≥ 3 times/week), physical activity (never, ≤ 2 times/week, or ≥ 3 times/week), socioeconomic status (low, middle, high), body mass index (BMI), fasting blood glucose level, systolic blood pressure (SBP), serum creatinine level, use of anti-thrombotic agents, use of anti-hypertensive agents according to class (renin–angiotensin–aldosterone system inhibitors, calcium channel blockers, beta blockers, alpha blockers, vasodilators, or diuretics), duration of diabetes, and baseline LDL-C levels (< 2.6, 2.6–3.3, ≥ 3.4 mmol/L).

All patients were followed-up from the first date of receiving statin therapy to the earliest occurrence of any cardiovascular outcomes described below, death, or the end of the cohort period (December 31, 2015). This study was approved by the Institutional Review Board of the Korea University Anam Hospital (IRB number ED17181). All data were anonymized; thus, the NHIS approved the study without requiring informed consent from each person.

### Outcome measures

The primary outcome measure was the occurrence of major adverse cardiovascular events (MACE) including ischemic heart disease (IHD), ischemic stroke (IS), or cardiovascular death. IHD was defined as hospitalization for IHD (identified by ICD-10 codes I20-I25) plus coronary artery angiography or procedures. IS was defined as hospitalization for IS (identified by ICD-10 code I63) plus brain imaging studies or procedures for IS. Cardiovascular death was defined as death from CVD (ICD codes I00-I99).

### Statistical analysis

Data are presented as mean and standard deviation (SD) for continuous variables and as number (n) and percentage (%) for categorical variables. A generalized estimating equation for matched data was used for the intergroup comparison (low-intensity group vs. moderate- or high-intensity group). For the primary outcome, we calculated incidence rates per 100 person-years and hazard ratios (HRs) with 95% confidence intervals (95% CIs) using Cox proportional hazard regression models. These analyses were performed according to statin intensity, achieved LDL-C concentration, and statin therapy duration. The achieved LDL-C concentration was defined as the minimum of LDL-C levels from 3 months after initiating statin therapy to the occurrence of outcomes or the end of follow-up. This was analyzed as a continuous variable, and the adjusted HR of MACE according to achieved LDL-C level was drawn using a cubic spline curve. Statin therapy duration was obtained by summing the length of a continuous statin prescription from the first day of study enrolment to the last day of follow-up. If the next prescription was filled within 30 days of the expected end date of the previous prescription, then therapy was considered uninterrupted. The duration of statin therapy was categorized into groups at intervals of 6 months: < 6, 6–12, 12–18, 18–24, 24–30, 30–36, and ≥ 36 months. The statin therapy duration was also analyzed as a continuous variable.

Furthermore, to compare the relative contributions of statin intensity, achieved LDL-C level, and statin therapy duration for predicting MACE, we calculated the proportion of log-likelihood explained by each factor. Log-likelihoods are useful for quantifying the predictive information contained in a variable compared with the information contained in the entire set of variables. The fraction of log-likelihood explained is analogous to R^2^ in an ordinary linear model. The partial effect of each risk factor was quantified by calculating the proportion of log-likelihood explained by each risk factor. A strong risk factor would contribute more, as compared with a weak risk factor, to the predictive ability of the model [18, 19]. The predictors included in the analyses were statin intensity, achieved LDL-C level, statin therapy duration, age, sex, socioeconomic status, duration of diabetes, BMI, fasting glucose, SBP, creatinine level, smoking, alcohol consumption status, exercise status, pre-existing CVD, and concurrent medication (anti-thrombotic agents, anti-hypertensive agents by class, and anti-diabetic agents by class). The statin therapy and diabetes duration were included as time-dependent variables.

All statistical analyses were performed using SAS software version 9.4 (SAS Institute Inc., Cary, NC, USA). Statistical significance was set at a two-sided p value < 0.05.

## Results

The baseline characteristics of the low-intensity and moderate- or high-intensity statin therapy groups were well balanced (Table 1). The mean age of the study participants was 63 years. Of them, 59.4% were male and 15% had pre-existing CVD. The mean LDL-C concentration was 3.3 mmol/L; 45.7% of the subjects had LDL-C levels of 3.4 mmol/L or higher, 33.6% of 2.6–3.3 mmol/L, and 20.7% of less than 2.6 mmol/L.

**Table 1:** Baseline characteristics of the participants after propensity score matching

	Low-intensity group (n = 383)	Matched control (1:1–27)	*P*-value^‡^
		Moderate- or high-intensity group	
		(n = 8554)	
Age (years)*	62.7 (9)	63 (9)	0.744
Male sex^†^	236 (62)	5075 (59)	0.631
SES^†^			0.280
Low	66 (17)	1664 (20)	
Moderate	107 (28)	2548 (30)	
High	210 (55)	4342 (51)	
Co-morbidities^†^			
Heart failure	10 (3)	219 (3)	0.956
IHD	19 (5)	468 (6)	0.896
IS	26 (7)	595 (7)	0.898
Current smoker^†^	75 (20)	1643 (19)	0.866
Current drinker^†^	153 (40)	3265 (38)	0.683
Regular exercise^†^	202 (53)	4637 (54)	0.623
Duration of diabetes (months)*	97.67 (46)	100.31 (45)	0.642
BMI (kg/m^2^)*	24.7 (3)	24.8 (3)	0.642
FBS (mmol/L)*	7.7 (2.7)	7.7 (2.6)	0.769
SBP (mmHg)*	129.7 (16)	129.8 (16)	0.927
Creatinine(umol/L)*	94.61 (88.4)	91.96 (88.4)	0.646
Anti-thrombotic agents^†^	165 (43)	3700 (43)	0.964
Anti-hypertensives agents^†^			
RAS inhibitor	196 (51)	4317 (51)	0.793
Calcium channel blocker	174 (45)	4014 (47)	0.732
β-blocker	119 (31)	2595 (30)	0.759
α-blocker	77 (20)	1645 (19)	0.740
Vasodilator	6 (2)	81 (1)	0.303
Diuretic	152 (40)	3508 (41)	0.795
Anti-diabetic agents^†^			
Metformin	347 (91)	7504 (88)	0.091
Sulfonylurea	266 (70)	6061 (71)	0.710
Thiazolidinedione	69 (18)	1415 (17)	0.428
DPP4-inhibitor	86 (23)	1674 (20)	0.181
Insulin	113 (30)	2354 (28)	0.376
Glinide	27 (7)	646 (8)	0.719
α-Glucosidase inhibitor	111 (29)	2324 (27)	0.391
LDL-C(mmol/L)^†^			0.169
< 2.6	86 (23)	1766 (21)	
2.6–3.3	140 (37)	2862 (34)	
≥ 3.4	157 (41)	3926 (46)	

Variables with * are presented as mean (standard deviation), while those with ^†^ are presented as number (%)

^‡^*P* value for intergroup comparison after propensity score matching by the generalized estimating equation method with appropriate specification of distribution and link function to each variable

SES: socioeconomic status; IHD: ischemic heart disease; IS: ischemic stroke; BMI: body mass index; FBS: fasting blood sugar; SBP: systolic blood pressure; RAS: renin–angiotensin–aldosterone system; DPP4: Dipeptidyl Peptidase-4; LDL-C: low-density lipoprotein cholesterol

Additional file 1: Table S1 shows the changes in the serum lipid profiles with statin therapy. The reduction of LDL-C levels (− 1.4 mmol/L vs − 1.1 mmol/L, *p* = 0.004) and non-HDL-C levels (− 1.6 mmol/L vs − 1.4 mmol/L, *p* = 0.001) was greater in the moderate- or high-intensity statin group than in the low-intensity statin group. The mean changes in HDL-C and TG levels were similar between groups.

### Statin intensity and risk of major cardiovascular events

A total of 861 major cardiovascular events occurred during follow-up (median, 41.9 months; interquartile range, 30.1–55.1 months). The incidence rate per 100 person-years was 3.76 in the low-intensity statin group versus 2.69 in the moderate- or high-intensity statin group. The risk of MACE was significantly lower in the moderate- or high-intensity statin group than in the low-intensity statin group (HR, 0.72; 95% CI 0.54–0.96) (Table 2 and Additional file 1: Fig S2). The risks of IHD, IS, and cardiovascular death were also lower in the moderate- or high-intensity statin group than in the low-intensity statin group, although the difference was not statistically significant.

**Table 2:** Cumulative incidence of major cardiovascular events by statin intensity

	Low-intensity group	Moderate- or high-intensity group	*P*-value†
	(n = 383)	(n = 8554)	
MACE			
No. of events	48	813	
Incidence rate*	3.76	2.69	
HR (95% CI)	1 (reference)	0.72 (0.54–0.96)	0.027
Ischemic heart disease			
No. of events	30	492	
Incidence rate*	0.1	1.6	
HR (95% CI)	1 (reference)	0.70 (0.49–1.01)	0.056
Ischemic stroke			
No. of events	17	293	
Incidence rate*	1.26	0.94	
HR (95% CI)	1 (reference)	0.75 (0.46–1.22)	0.240
Cardiovascular death			
No. of events	5	94	
Incidence rate*	0.36	0.3	
HR (95% CI)	1 (reference)	0.82 (0.33–2.01)	0.659

^*^Incidence rate per 100 person-years

^†^*P* value by Cox proportional hazards model with robust variance estimator for clustered data

HR: hazard ratio; CI: confidence interval; MACE: major adverse cardiovascular event

### Achieved LDL-C Level and risk of major cardiovascular events

**Fig. 1:**
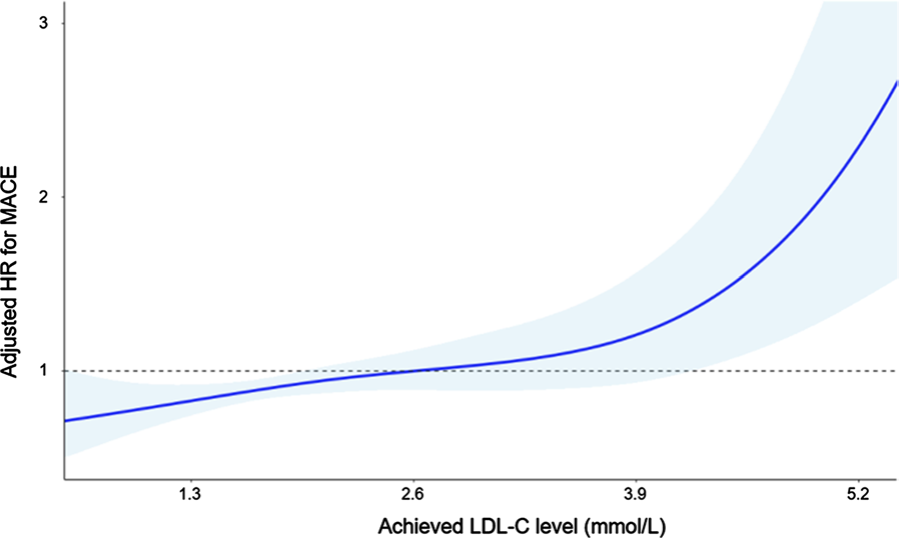
Cubic spline curve of adjusted hazard ratio for major cardiovascular events according to achieved LDL-C levels (mmol/L). Adjusted HR* (blue line) and 95% CI (shaded area) for major cardiovascular events according to on-treatment LDL-C levels with a reference of 2.6 mmol/L. The achieved LDL-C levels of the points at which 95% CI meets the reference line (HR = 1) are 1.9 mmol/L and 4.2 mmol/L. *Adjusted for age, sex, socioeconomic status, duration of diabetes, body mass index, fasting blood glucose, systolic blood pressure, creatinine, smoking, alcohol consumption, exercise, pre-existing cardiovascular disease (ischemic heart disease, ischemic stroke, and heart failure), and concurrent medications (anti-thrombotic agents, anti-hypertensive agents by class, and anti-diabetic agents by class)

Given that the risk of MACE was determined by statin intensity, further analyses were performed with patients treated with moderate- or high-intensity statins (n = 8554). We evaluated the association between achieved LDL-C levels and the risk of MACEs. In the cubic spline curve analysis with achieved LDL-C as a continuous variable, patients with lower LDL-C levels had a lower cardiovascular risk (Fig. 1).

### Statin duration and risk of major cardiovascular events

**Fig. 2:**
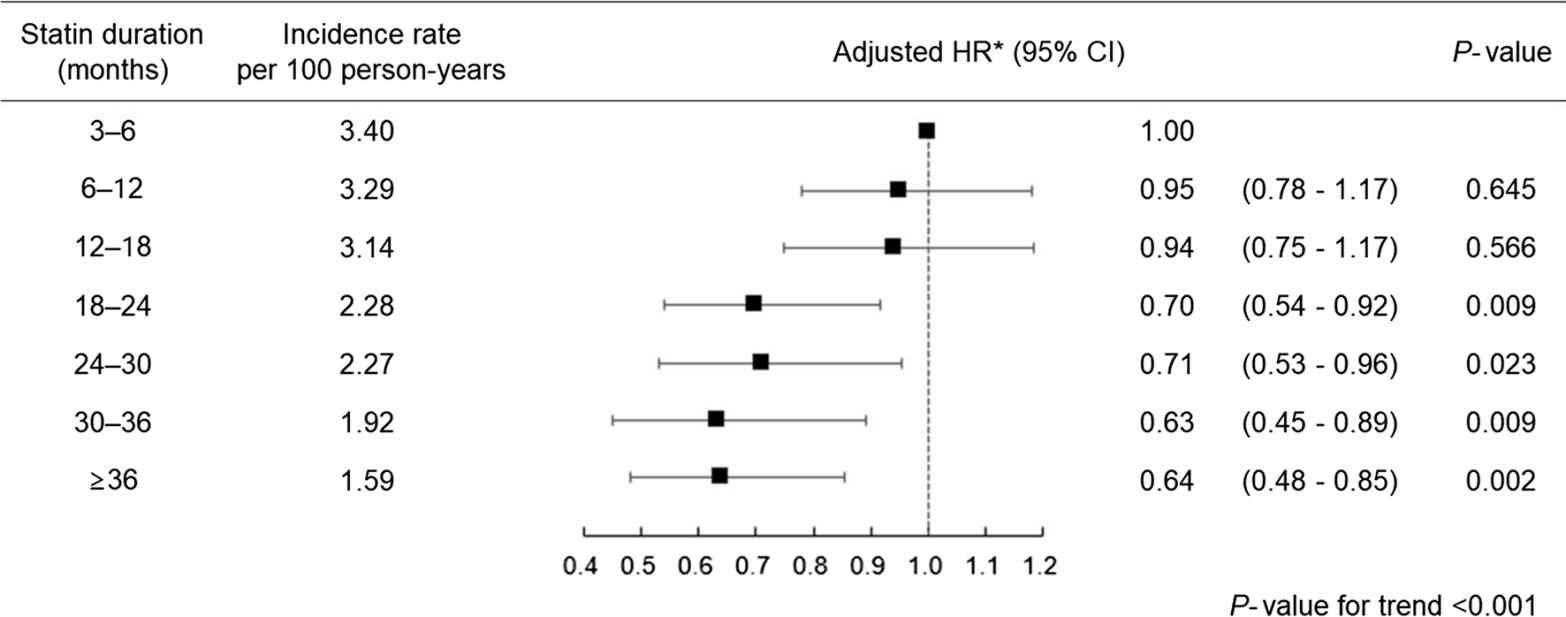
Risk of major cardiovascular events according to statin therapy duration in patients who received moderate- or high-intensity statins. *Adjusted for age, sex, socioeconomic status, duration of diabetes, body mass index, fasting blood glucose, systolic blood pressure, creatinine, smoking, alcohol consumption, exercise, pre-existing cardiovascular disease (ischemic heart disease, ischemic stroke, and heart failure), concurrent medications (anti-thrombotic agents, anti-hypertensive agents by class, and anti-diabetic agents by class), and pre-treatment LDL-C level

**Fig. 3:**
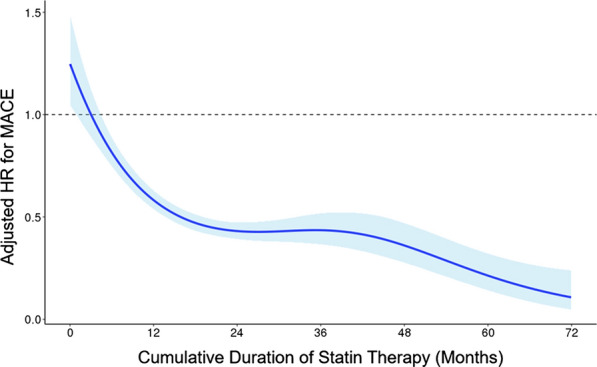
Cubic spline curve of adjusted hazard ratio for major cardiovascular events according to statin therapy duration. Adjusted HR* (solid line) and 95% CI (shaded area) for major cardiovascular events according to statin therapy duration were calculated with a reference of 3 months. *Adjusted for age, sex, socioeconomic status, duration of diabetes, body mass index, fasting blood glucose, systolic blood pressure, creatinine, smoking, alcohol consumption, exercise, pre-existing cardiovascular disease (ischemic heart disease, ischemic stroke, and heart failure), and concurrent medications (anti-thrombotic agents, anti-hypertensive agents by class, and anti-diabetic agents by class), and pre-treatment LDL-C level

We estimated the HRs of MACEs according to the statin therapy duration. First, the adjusted HRs of MACE were calculated by categorizing the duration of statin therapy. Compared to patients who received statin therapy for less than 6 months, the risk of MACE was gradually reduced with an increase in statin therapy duration. Adjusted HRs were 0.70 (95% CI 0.54–0.92), 0.71 (95% CI 0.53–0.96), 0.63 (95% CI 0.45–0.89), and 0.64 (95% CI 0.48–0.85) for patients who received statin therapy for 18–24, 24–30, 30–36, and ≥ 36 months, respectively (Fig. 2). The duration of statin use was also analyzed as a continuous variable. Figure 3 shows the cubic spline curve of the adjusted HR according to statin therapy duration. The longer the patients received statins, the lower was the risk of MACE.

### Relative importance of statin intensity, achieved LDL-C level, and statin therapy duration

**Fig. 4:**
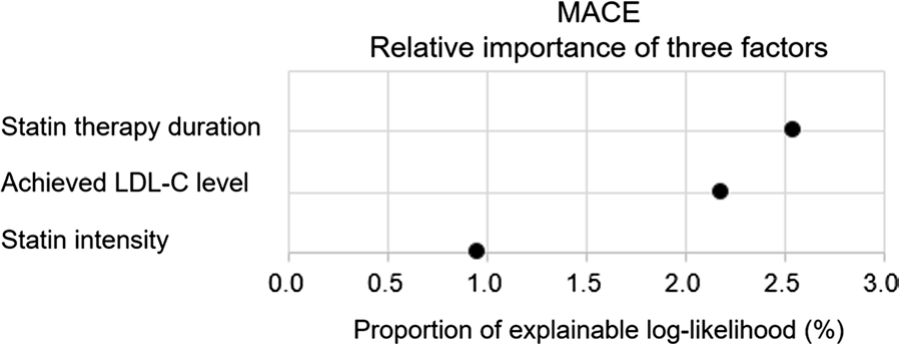
Relative importance of statin therapy duration, statin intensity, and achieved LDL-C levels as predictors for MACE. The relative contributions of statin therapy duration, statin intensity, and achieved LDL-C levels were quantified by calculating the proportion of log-likelihood explained by each risk factor. Other variables in the model included age, sex, socioeconomic status, duration of diabetes, body mass index, fasting blood glucose, systolic blood pressure, creatinine, smoking, alcohol consumption, exercise, pre-existing cardiovascular disease (ischemic heart disease, ischemic stroke, and heart failure), and concurrent medication (anti-thrombotic agents, anti-hypertensive agents by class, and anti-diabetic agents by class). The statin therapy and diabetes durations were included as time-dependent variables

Given that statin intensity, achieved LDL-C, and statin therapy duration were all determining factors for the risk of cardiovascular events, we compared the relative importance of these factors. The relative contributions were estimated by computing the proportion of explainable log-likelihood explained by each factor; the strongest predictor of the risk of MACE was statin therapy duration (2.55), followed by achieved LDL-C level (2.18) and statin intensity (0.95) (Fig. 4 and Additional file 1: Table S2).

## Discussion

In this study, we found that all three components of statin therapy—statin intensity, achieved LDL-C level, and statin therapy duration—significantly affected cardiovascular risks in T2D patients. Higher statin intensity, lower achieved LDL-C, and longer statin therapy duration reduced the risk of MACE. When all factors were included in a model for assessing cardiovascular risk, the statin therapy duration was even more important than statin intensity or achieved LDL-C level.

It is well established that apolipoprotein B (ApoB)-containing lipoproteins, including LDL, are implicated in the initiation and progression of atherosclerotic changes in the vasculature [20]. Statins exert their effects mainly by increasing LDL clearance from the blood by upregulating LDL receptors [21]. Numerous RCT and meta-analyses confirmed cardiovascular risk reduction with statins in multiple patient groups at different levels of cardiovascular risk [3–5, 22]. Based on previous RCT and meta-analyses, current guidelines for dyslipidemia management generally recommend the use of moderate- or high-intensity statins in high-risk patients, including those with T2D [6, 23]. However, controversy persists regarding whether a lower LDL-C level is better. In a population-based cohort study, achieving an LDL-C of 1.8 mmol/L or less with statin therapy did not have additional benefit over a level of 2.6 mmol/L or less in patients with IHD [8]. Although recent trials of proprotein convertase subtilisin/kexin 9 (PCSK9) inhibitors and ezetimibe strengthened the concept of “lower is better,” most of those trials included very high-risk groups [24–26]. Our study provides evidence that the “lower is better” concept would be applicable to T2D patients with diverse cardiovascular risk. There was no threshold for LDL-C concentrations for discriminating cardiovascular risk. Considering that only a small proportion of patients receiving statins achieved LDL-C targets and late initiation of high-intensity statin appears to be pivotal factors needing to be modified for improving CVD prevention [27] and that primary prevention statin therapy remains underutilized [28], clinicians should be more aggressive for lowering LDL-C levels even in patients without pre-existing CVD.

Besides the LDL-C lowering effects, statins have shown pleiotropic effects, such as inhibition of vascular inflammation, immune modulation, effects on endothelial progenitor stem cells, and effects on thrombogenicity [29]. Especially, higher intensity statin therapy has shown improvements in other pro-atherogenic factors and LDL-related parameters such as non-HDL-C and ApoB [30]. Our study also showed that moderate- or high-intensity statin reduced non-HDL-C more than low-intensity statin did. This could be contributed to greater cardiovascular risk reduction of higher intensity statins as well.

Despite a large number of patients discontinuing statin therapy in clinical practice [9, 31, 32], the effect of statin non-adherence on cardiovascular risk reduction has not been sufficiently studied. We revealed that the constant use of statins was an important factor in determining the cardioprotective effect of statin therapy. Specifically, at least 18 months of statin therapy was associated with a significantly lower risk of MACE compared to less than 6 months of statin therapy. Notably, the longevity of statin use was more important than other statin therapy factors for determining their cardioprotective effects. We suppose that the continued use of statins is critical in reducing exposure to ApoB-containing lipoproteins, which increases an individual’s total atherosclerotic plaque burden. This hypothesis was clearly proven in the spline curve analysis with statin therapy duration as a continuous variable. Recently, it has been reported that inpatient statin use and continuous use of statin was associated with lower in-hospital mortality compared to no statin use and discontinuation of statins in patients with coronavirus disease 2019 [33, 34]. Plausible explanations for these benefits of statins include anti-inflammatory and immunomodulating properties of statins, which may also be associated with lowering cardiovascular risk. Overall, the continuous use of statin should be emphasized in clinical practice.

This study has several limitations. First, due to the retrospective study design, the analyses established only associations between risk factors and outcomes but could not determine any causal relationship. Although we tried to adjust for confounding variables, there might be unmeasured confounders such as the individual’s health consciousness, eating habits and adherence to other medications. Second, we could not adjust for some important variables, including glycated hemoglobin, due to the lack of relevant data. Instead, the average fasting blood glucose was adjusted. Third, the number of patients in the low-intensity group was much smaller than that in the moderate- or high-intensity group. Lastly, LDL-C-lowering medications other than statins were not considered since ezetimibe was not used widely and PCSK9 inhibitors were not approved in South Korea during the study period. Further investigations are required to consider the effects of these agents.

## Conclusions

In conclusion, this population-based study suggested that statin therapy duration or adherence should be considered an important factor for cardioprotective effects of statins in clinical practice. We propose the concept “longer is better” for statin therapy rather than “lower is better” for target LDL-C levels.

## Supplementary Information

**Additional file 1: Table S1.** Pre- and post-treatment lipid profiles with statin therapy. **Table S2.** Relative importance of predictors for MACE by estimating the log-likelihood explained by each predictor. **Figure S1.** Flow diagram of the study subject selection process. **Figure S2.** Cumulative incidence of major cardiovascular events by statin intensity.

## Data Availability

Additional data are available through approval and oversight by the Korean National Health Insurance Service (available at https://nhiss.nhis.or.kr).

## Abbreviations

ApoB: Apolipoprotein B
BMI: Body mass index
CI: Confidence interval
CVD: Cardiovascular disease
HDL-C: High-density lipoprotein cholesterol
HR: Hazard ratio
ICD-10: International Classification of Disease, Tenth Revision
IHD: Ischemic heart disease
IS: Ischemic stroke
LDL-C: Low-density lipoprotein cholesterol
MACE: Major adverse cardiovascular event
NHIS-HEALS: National Health Insurance Service-Health Screening Cohort
PCSK9: Proprotein convertase subtilisin/kexin 9
RCT: Randomized controlled trial
SBP: Systolic blood pressure
SD: Standard deviation
T2D: Type 2 diabetes
TG: Triglyceride
