# Supplementary material for: Relative contributions of statin intensity, achieved low-density lipoprotein cholesterol level, and statin therapy duration to cardiovascular risk reduction in patients with type 2 diabetes: population based cohort study
Source: Cardiovasc Diabetol. 2022 Feb 22;21:28. doi: 10.1186/s12933-022-01466-z (PMC8861991; doi:10.1186/s12933-022-01466-z)

**Table S1.** Pre- and post-treatment lipid profiles with statin therapy

|  |  |  | Matched control (1:1–27) | | *P-* value* |
| --- | --- | --- | --- | --- | --- |
|  | Low-intensity group | | Moderate- or high-intensity group | |  |
|  | (n = 383) | | (n = 8554) | |  |
| LDL-C (mmol/L) |  |  |  |  |  |
| Pre-treatment | 3.23 | (0.92) | 3.29 | (0.92) | 0.754 |
| Post-treatment | 2.03 | (0.67) | 1.93 | (0.66) |  |
| Change | -1.13 | (1.03) | -1.35 | (0.89) | 0.004 |
| HDL-C (mmol/L) |  |  |  |  |  |
| Pre-treatment | 1.26 | (0.38) | 1.31 | (0.35) | 0.017 |
| Post-treatment | 1.28 | (0.31) | 1.30 | (0.33) |  |
| Change | -0.00 | (0.41) | -0.02 | (0.34) | 0.553 |
| Triglyceride (mmol/L) |  |  |  |  |  |
| Pre-treatment | 2.00 | (1.21) | 2.01 | (1.24) | 0.776 |
| Post-treatment | 1.55 | (0.89) | 1.48 | (0.86) |  |
| Change | -0.44 | (1.06) | -0.51 | (1.11) | 0.352 |
| Total cholesterol (mmol/L) |  |  |  |  |  |
| Pre-treatment | 5.40 | (1.02) | 5.52 | (1.02) | 0.096 |
| Post-treatment | 3.98 | (0.80) | 3.90 | (0.79) |  |
| Change | -1.36 | (1.08) | -1.61 | (0.96) | 0.001 |
| Non-HDL-C (mmol/L) |  |  |  |  |  |
| Pre-treatment | 4.14 | (0.98) | 4.21 | (0.97) | 0.453 |
| Post-treatment | 2.71 | (0.74) | 2.61 | (0.73) |  |
| Change | -1.36 | (1.10) | -1.59 | (0.94) | 0.001 |

Data are presented as mean (standard deviation).

**P* value calculated using the generalized estimating equation method.

LDL-C, low-density lipoprotein cholesterol; HDL-C, high-density lipoprotein cholesterol.

**Table S2**. Relative importance of predictors for MACE by estimating the log-likelihood explained by each predictor

| Order  of importance | Variables | Proportion of explainable log-likelihood (%) |
| --- | --- | --- |
| 1 | Pre-existing IHD | 12.14 |
| 2 | Insulin | 4.65 |
| 3 | Sex | 4.57 |
| 4 | Pre-existing IS | 4.28 |
| 5 | Statin therapy duration* | 2.55 |
| 6 | Age | 2.39 |
| 7 | Achieved LDL-C level | 2.18 |
| 8 | Anti-thrombotic agent | 1.82 |
| 9 | Sulfonylurea | 1.45 |
| 10 | Statin intensity | 0.95 |
| 11 | Pre-existing heart failure | 0.80 |
| 12 | Smoking | 0.72 |
| 13 | Alcohol consumption | 0.56 |
| 14 | Systolic blood pressure | 0.47 |
| 15 | Vasodilator | 0.44 |
| 16 | α-glucosidase inhibitor | 0.42 |
| 17 | Body mass index | 0.39 |
| 18 | Duration of diabetes* | 0.37 |
| 19 | α-blocker | 0.37 |
| 20 | Calcium channel blocker | 0.33 |
| 21 | RAS inhibitor | 0.31 |
| 22 | Metformin | 0.22 |
| 23 | Socioeconomic status | 0.18 |
| 24 | Regular exercise | 0.17 |
| 25 | DPP4-inhibitor | 0.10 |
| 26 | β-blocker | 0.06 |
| 27 | Diuretic | 0.03 |
| 28 | Thiazolidinedione | 0.01 |
| 29 | Glinide | 0.01 |
| 30 | Fasting blood glucose | 0.01 |
| 31 | Creatinine | <0.01 |

The relative contributions of predictors for MACE were quantified by calculating the proportion of explainable log-likelihood explained by each risk factor.

*The statin therapy and diabetes durations were included as time-dependent variables.

MACE, major adverse cardiovascular event; IHD, ischemic heart disease; IS, ischemic stroke; LDL-C, low-density lipoprotein cholesterol; RAS, renin–angiotensin–aldosterone; DPP4, Dipeptidyl Peptidase-4.

**Figure S1**. Flow diagram of the study subject selection process.


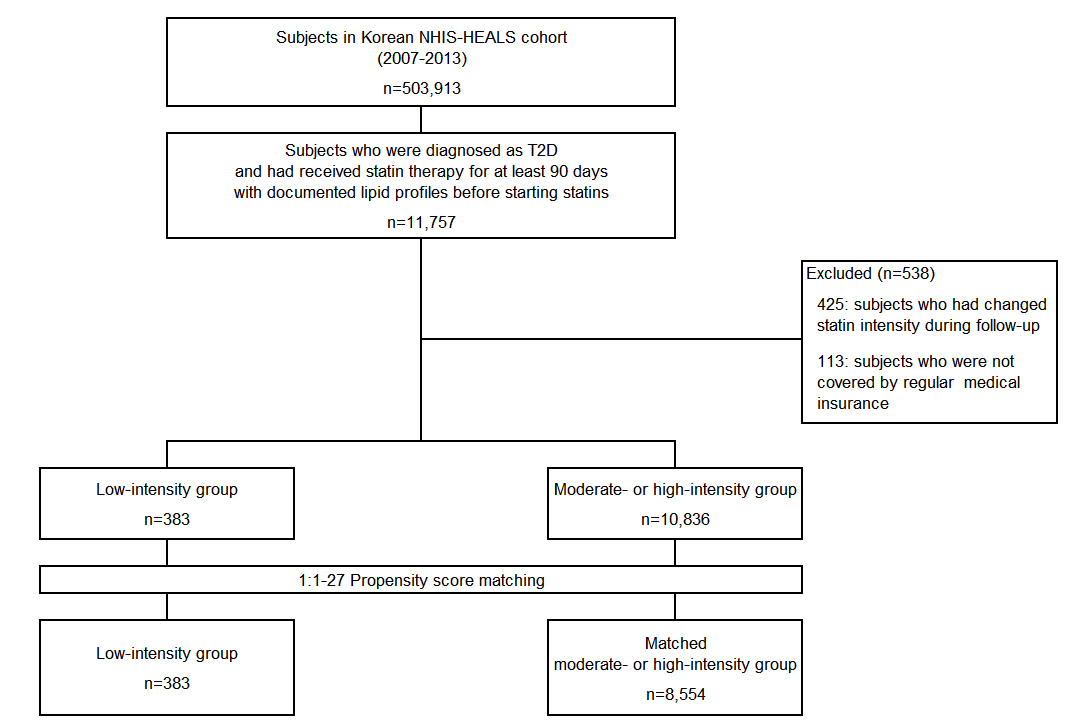


**Figure S2.** Cumulative incidence of major cardiovascular events by statin intensity


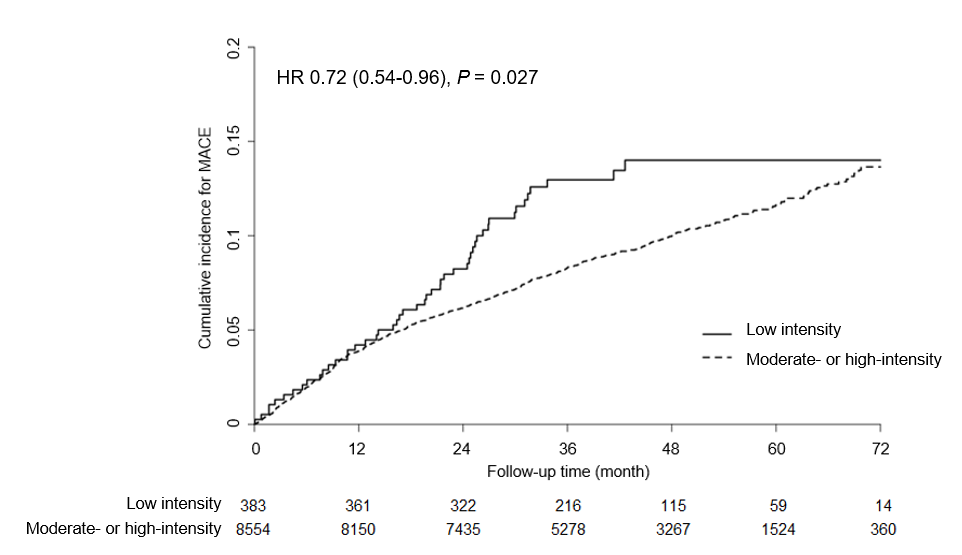

Supplement: Supplementary file 1 — Additional file 1: Table S1. Pre- and post-treatment lipid profiles with statin therapy. Table S2. Relative importance of predictors for MACE by estimating the log-likelihood explained by each predictor. Figure S1. Flow diagram of the study subject selection process. Figure S2. Cumulative incidence of major cardiovascular events by statin intensity. [file 12933_2022_1466_MOESM1_ESM.docx]
